# Gestational intermittent hyperoxia rescues murine genetic congenital heart disease in part

**DOI:** 10.1038/s41598-021-85569-9

**Published:** 2021-03-23

**Authors:** Cassandra F. Doll, Natalia J. Pereira, Mustafa S. Hashimi, Tabor J. Grindrod, Fariz F. Alkassis, Lawrence X. Cai, Una Milovanovic, Adriana I. Sandino, Hideko Kasahara

**Affiliations:** 1 Department of Physiology and Functional Genomics, University of Florida College of Medicine, 1600 SW Archer Rd. M543, Gainesville, FL 32610-0274 USA; 2 International University of Health and Welfare, School of Medicine, 852 Hatakeda, Narita, Chiba Japan

**Keywords:** Cardiology, Diseases

## Abstract

Cardiac development is a dynamic process, temporally and spatially. When disturbed, it leads to congenital cardiac anomalies that affect approximately 1% of live births. Genetic variants in several loci lead to anomalies, with the transcription factor *NKX2-5* being one of the largest. However, there are also non-genetic factors that influence cardiac malformations. We examined the hypothesis that hyperoxia may be beneficial and can rescue genetic cardiac anomalies induced by an *Nkx2-5* mutation. Intermittent mild hyperoxia (40% PO_2_) was applied for 10 h per day to normal wild-type female mice mated with heterozygous *Nkx2-5* mutant males from gestational day 8.5 to birth. Hyperoxia therapy reduced excessive trabeculation in *Nkx2-5* mutant mice compared to normoxic conditions (ratio of trabecular layer relative to compact layer area, normoxia 1.84 ± 0.07 vs. hyperoxia 1.51 ± 0.04) and frequency of muscular ventricular septal defects per heart (1.53 ± 0.32 vs. 0.68 ± 0.15); however, the incidence of membranous ventricular septal defects in *Nkx2-5* mutant hearts was not changed. *Nkx2-5* mutant embryonic hearts showed defective coronary vessel organization, which was improved by intermittent mild hyperoxia. The results of our study showed that mild gestational hyperoxia therapy rescued genetic cardiac malformation induced by *Nkx2-5* mutation in part.

## Introduction

Congenital cardiac anomalies are the most prevalent birth defects, affecting approximately 1% of live births^1–3^. Genetic variants in several loci lead to anomalies^4–6^. Among these, heterozygous homeodomain-containing transcription factor *NKX2-5* variants are one of the largest sets (OMIM, NCBI), and currently, nearly 40 variants have been reported in humans^7,8^. The same nucleotide variant can lead to varied types and severity of disease^9^, not only in humans, but also in *Nkx2-5*^+/R52G^ knock-in mutant mice (hereafter called *Nkx2-5* mutant) that have the same genetic background through backcrossing^8,10,11^. These observations strongly suggest non-genetic factors have influence on cardiac malformations. In addition, the majority of congenital cardiac anomalies cannot be linked to specific genes^9,12^, suggesting that non-genetic factors alone can lead to cardiac anomalies.

Gestational hypoxia increases the risk of low intrauterine growth, low birth weight, and developing cardiovascular defects. Various conditions such as high-altitude pregnancies, maternal smoking, congestive heart failure, pulmonary diseases, acute/chronic respiratory tract infections, anemia, preeclampsia, and placental insufficiency cause gestational hypoxia^13^. Gestational hypoxia alone can induce cardiac anomalies in experimental animal models^14–17^. Consistently, in our experiments, when wild-type pregnant mice were housed under moderate chronic hypoxia (14% of O_2_) conditions from gestational day 10.5 until term, newborn mice showed cardiac anomalies such as excessive trabeculation, ventricular septal defects (VSDs), irregular morphology of interventricular septum as well as atrial septal abnormalities, which overlap with those seen in heterozygous *Nkx2-5* mutant mice^18^. In addition, genome-wide transcriptome done by RNA-seq of a 2-day hypoxic exposure on wild-type embryos revealed abnormal transcriptomes in which approximately 60% share those from *Nkx2-5* mutants without hypoxia. Gestational hypoxia reduced the expression of Nkx2-5 proteins in more than one-half, suggesting that abnormal Nkx2-5 function is a common mechanism shared between genetic and gestational hypoxia-induced cardiac anomalies^18^. In heterozygous *Nkx2-5* knockout mice, Nkx2-5 expression was also reduced by severe hypoxia at the transcriptional level rather than at the translational level^17^.

Advances in clinical care such as surgical interventions have enabled most patients with congenital heart disease to reach adulthood. This prolonged survival, however, has been achieved at a cost, as many patients suffer later complications, of which heart failure and arrhythmias are the most prominent^12^. Alternative treatment aimed at correcting abnormally developing hearts may offer potential in the future^8,19^. Maternal hyperoxygenation has been investigated as a possible therapy for augmenting ventricular growth in fetuses with congenital heart disease, in particular with underdeveloped cardiac chambers (reviewed in Refs.^19,20^). The therapeutic potential of hyperoxia also has been tested in rodents with cardiac and noncardiac anomalies, including coarctation of aorta^21^, hypoplastic cardiovascular structure^22^, cleft lip^23^, and phenytoin-induced cleft palate^24^.

High levels of oxygen (i.e., above 80% PO_2_) has adverse effects such as inducing bronchopulmonary dysplasia, retinopathy, prematurity and perinatal brain injury^25–27^. In this study, we applied mild hyperoxia, and have demonstrated that intermittent hyperoxia (40% of O_2_, 10 h per day) starting from gestational day 8.5 partly rescued cardiac anomaly in terms of reducing the frequencies of muscular VSD and ventricular non-compaction with correction of defective coronary vessel organization in *Nkx2-5* mutant hearts.

## Results

### Micro-computed tomography (CT) imaging is an alternative to serial paraffin-embedded tissue sectioning for detecting cardiac anomalies in postnatal day 1 (P1) mice

**Figure 1:**
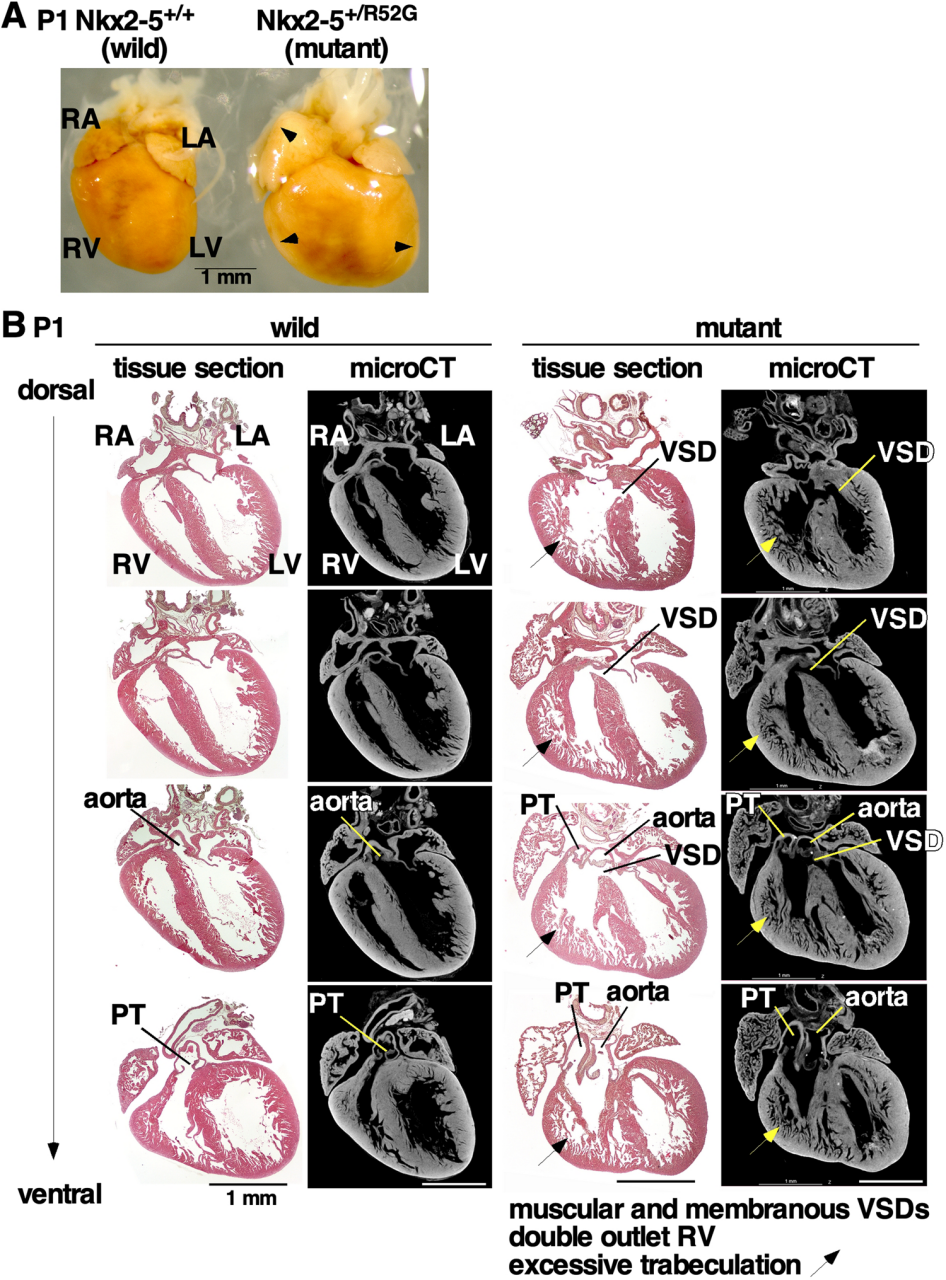
Representative cardiac anomaly in postnatal day 1 *Nkx2-5*^+*/R52G*^ mouse and control wild-type littermate. Control *Nkx2-5*^+*/*+^ (left) vs. *Nkx2-5*^+*/R52G*^ (right): (**A**) dissected hearts, (**B**) heart tissue sections and corresponding micro-computed tomography images. Excessive trabeculation in *Nkx2-5*^+*/R52G*^ is indicated by arrows. *LA* left atrium, *LV* left ventricle, *PT* pulmonary trunk, *RA* right atrium, *RV* right ventricle, *VSD* ventricular septal defect.

Micro-CT has been developing as an alternative method to serial paraffin-embedded tissue sectioning to obtain quantitative imaging of cardiac anomalies. Iodine contrast-enhanced micro-CT scan with 4-µm resolution followed by three-dimensional (3D) reconstruction was applied to heterozygous P1 *Nkx2-5* mutant hearts showing varied cardiac anomalies as reported previously^11^. An example of an *Nkx2-5* mutant heart grossly enlarged with expectations of complicated cardiac anomalies was chosen to examine the feasibility and ability of micro-CT imaging (Fig. 1A). Serial paraffin-embedded tissue sectioning of 5 µm thickness and micro-CT scanned images side-by-side were comparable and both demonstrated muscular and peri-membranous VSD, double outlet of right ventricle and excessive trabeculation in *Nkx2-5* mutant heart (Fig. 1B, tissue section vs. micro-CT). Notably, optical sectioning of micro-CT imaging can be done from any axes, which improves a quality of diagnosis of cardiac anomalies relative to physical histology tissue sectioning (see Supplemental Video S1).

### Intermittent mild hyperoxia (40% O_2_) for 10 h per day decreases a number of muscular VSD per heart and the degree of ventricular non-compaction in *Nkx2-5* mutant mice

We recently demonstrated that continuous gestational mild hypoxia (14% PO_2_) induces cardiac anomalies in wild-type mice, including excessive trabeculation and peri-membranous and muscular VSDs, which partly overlap with those seen in heterozygous *Nkx2-5* mutant mice^18^. As opposed to hypoxia, hyperoxia may be effective for rescuing *Nkx2-5* mutant mice in part.

**Figure 2:**
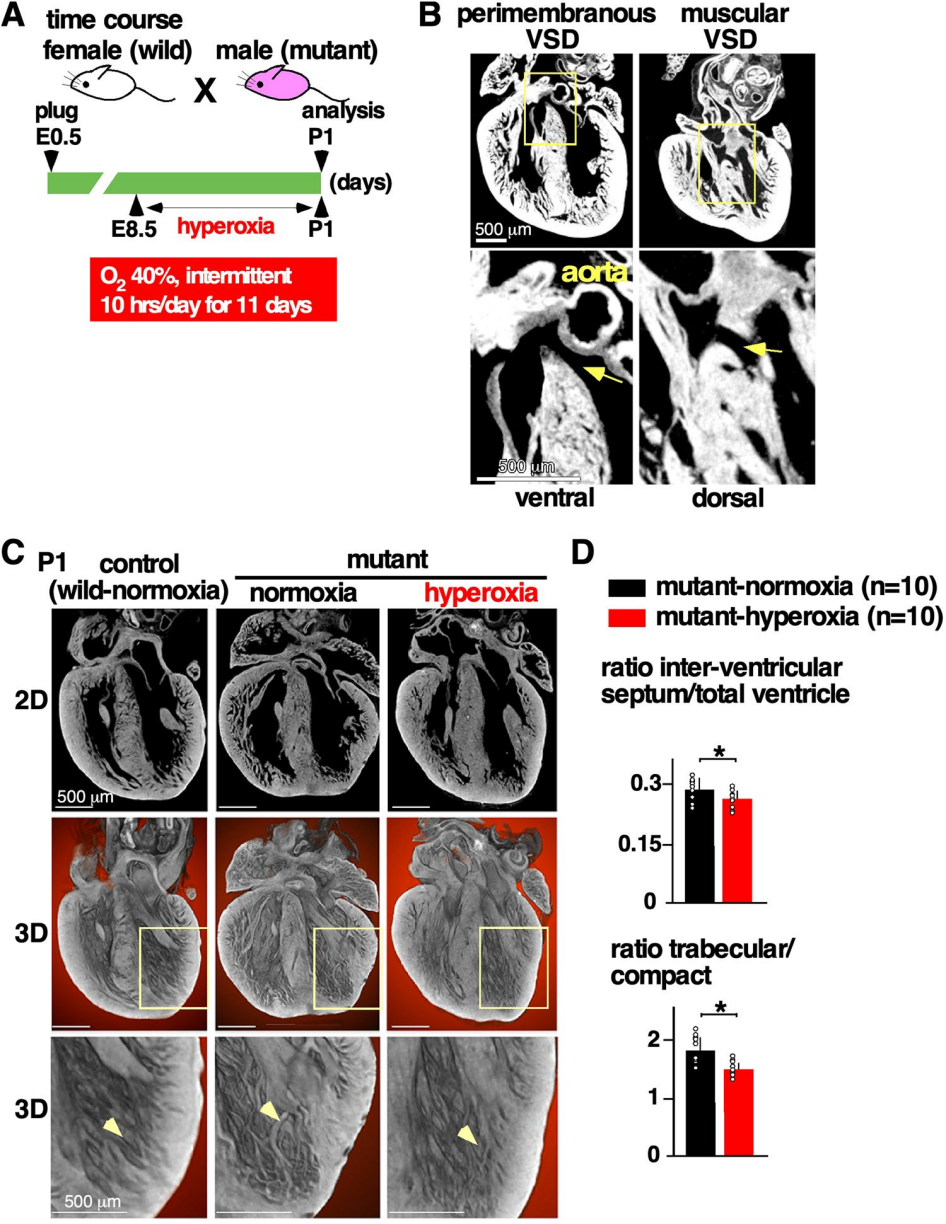
Experimental design of gestational hyperoxia (40% O_2_ saturation) and representative images of postnatal day 1 *Nkx2-5* mutant hearts using micro-computed tomography (CT). (**A**) Timeline of experiment. (**B**) Representative 2D images demonstrating perimembranous and muscular VSD regarding their location (arrows). (**C**) Representative 2D and 3D micro-CT images showing ventricular trabeculation (arrowheads). (**D**) Quantification of area size of total ventricle, inter-ventricular septum, trabecular and compact layers. Data are expressed as mean ± S.D.; individual data are represented as circles. The Shapiro–Wilk normality test confirmed normal distribution of variables. *P ≤ 0.05. *VSD* ventricular septal defect. ImageJ 1.52d (http://imagej.nih.gov/ij) was used for analysis.

In consideration of the toxicity of high levels of oxygen (i.e., above 80% PO_2_)^25–27^, and the difficulty in applying continuous 24 h hyperoxia to pregnant humans with the understanding that this interferes with normal activities of daily living (bathing, meals, and work, etc.), we applied an intermittent mild hyperoxia (40% PO_2_, 10 h per day). Normal wild-type females mated with heterozygous *Nkx2-5* mutant males were housed in the hyperoxia chamber during the daytime, which is considered the night in humans, from the gestational day 8.5 to birth (Fig. 2A). Control mice were housed under normoxic conditions. P1 mouse hearts were analyzed using micro-CT two-dimensional (2D) and three-dimensional (3D) imaging.

The most prevalent lesions seen in *Nkx2-5* mutant hearts were excessive trabeculations and perimembranous and/or muscular VSDs found in a single or multiple positions^11^. An example of an *Nkx2-5* mutant heart showed a perimembranous VSD lacking fibrous continuity between the developing leaflets of the aortic and tricuspid valves, and an additional muscular defect positioned dorsally, being enclosed within the musculature of the septum (Fig. 2B). Mild hyperoxia rescued cardiac anomalies in part by reducing a number of multiple muscular VSDs per heart compared to normoxic conditions (1.53 ± 0.32 vs. 0.68 ± 0.15); however, an incidence of peri-membranous or muscular VSD was not reduced (Table 1).

**Table 1:** Summary of cardiac anomalies of P1 *Nkx2-5* mutant mice with or without hyperoxia.

Phenotypes	Mutant-normoxia (n = 19)	Mutant-hyperoxia (n = 22)	*P* value
Membranous VSD	6 (31.6%)	7 (31.8%)	0.626
Muscular VSD	13 (68.4%)	12 (54.5%)	0.279
Number of muscular VSDs per heart	1.53 ± 0.32	0.68 ± 0.15	0.024*

The one-sided Fisher’s exact test was used on a 2 × 2 contingency table for calculation of *P* values for incidence of membranous or muscular VSDs.

*P ≤ 0.05.

Regarding another feature of *Nkx2-5* mutant hearts, namely excessive trabeculations, we quantified the total area of the trabecular and compact layers of the walls and interventricular septum areas and compared the relative ratio as described^11^. We first excluded hearts that demonstrated profound anomalies, which obviously changes the ventricular size and morphology, such as double outlets of right ventricle shown in Fig. 1, and then we randomly selected 10 hearts from each group. Although the discrimination between the compact and trabecular layers can be somewhat subjective, we followed consistent criteria throughout the analyses as described in the Materials and Methods. Mild hyperoxia reduced interventricular septum areas relative to the total ventricular area and trabecular layer relative to compact layer area compared to normoxic condition (Fig. 2C,D).

### Defective coronary vessel organization in *Nkx2-5* mutant embryonic hearts was improved by intermittent mild hyperoxia

The initiation of compact layer thickening (outer layer) and the formation of coronary vasculature occur in an interdependent manner around E11-12^28–30^. Around E11.5, endothelial cells in the coronary vasculature in the ventricular wall are thought to originate from the sinus venosus localized on the dorsal surface of the myocardium, and migrate between the epicardium and myocardium^31–35^. Because *Nkx2-5* mutant hearts show excessive trabeculation, we examined whether coronary vessel organization may be defective in *Nkx2-5* mutant mice.

**Figure 3:**
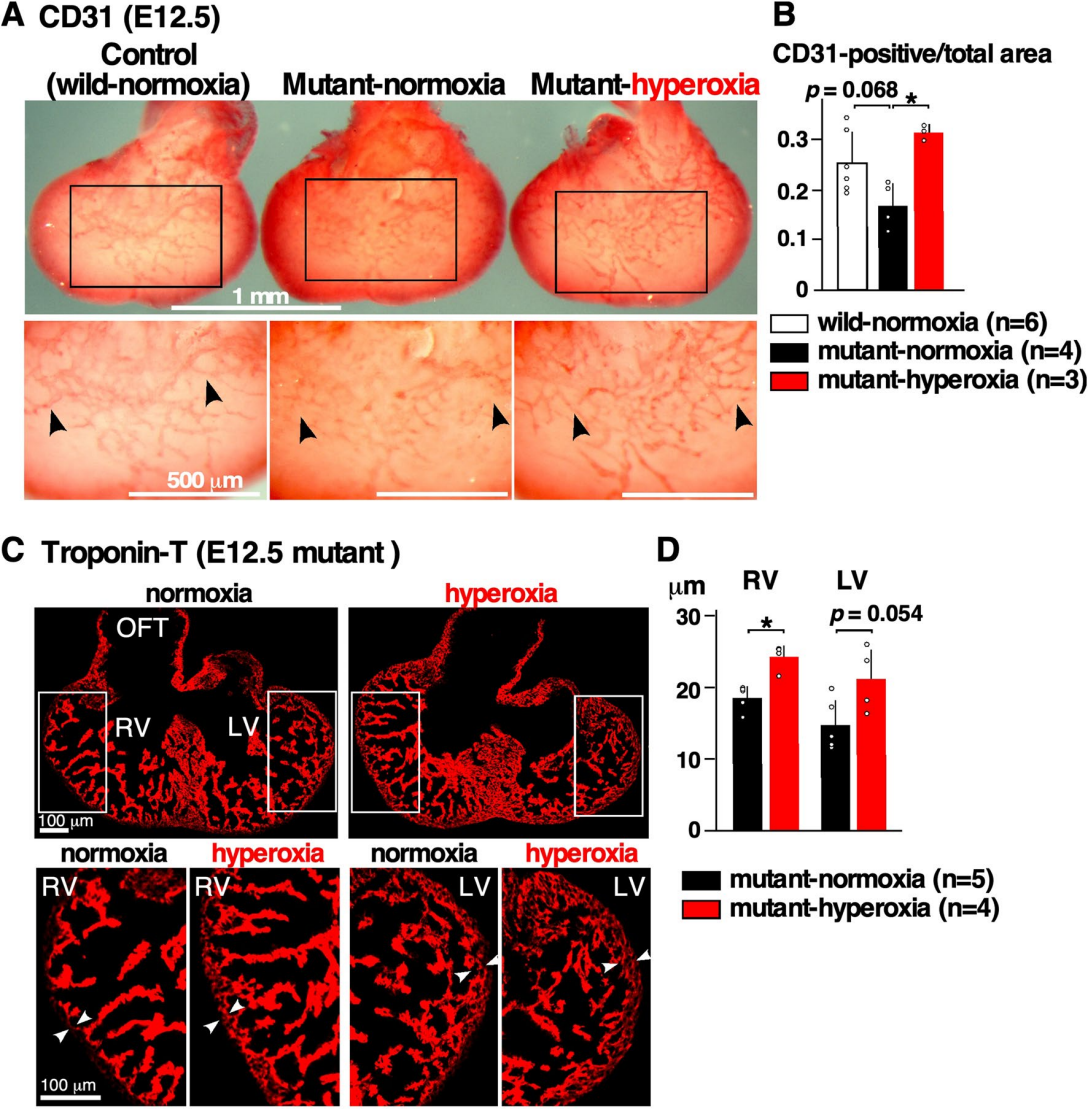
Defective coronary vessel organization in E12.5 *Nkx2-5* mutant embryonic hearts, and the effects of hyperoxia. (**A**) Dorsal view of whole-mount CD31 immunostaining of ventricles showing endothelial cell plexus organized within the ventricles. CD31 staining was less prominent and extended to the apical area in *Nkx2-5* mutant heart at normoxia, but was comparable at hyperoxia to the control wild-type heart (black arrowheads). (**B**) Quantitative CD31-positive area relative to the entire ventricular area. The number of embryos examined is indicated. The non-parametric Kruskal–Wallis ANOVA with Dunn’s test was used for analysis. (**C**) Representative heart sections stained with troponin-T. Thickness of compact layer is shown in white arrowheads. (**D**) Quantitative data showing thickness of compact layer in *Nkx2-5* mutant hearts at normoxia vs. hyperoxia. The Mann–Whitney U test was used for analysis. Data are expressed as mean ± S.D.; individual data are represented as circles. *P ≤ 0.05. *LV* left ventricle, *RV* right ventricle, *OFT* outflow tract. The number of mice examined is indicated. ImageJ 1.52d (http://imagej.nih.gov/ij) was used for analysis.

Coronary vessel organization has not been examined previously in *Nkx2-5* mutant hearts, thus we first compared it between normoxic wild-type and *Nkx2-5* mutant developing hearts using whole-mount immunostaining with the endothelial marker CD31 (Fig. 3A, wild-normoxia vs. mutant-normoxia). Dorsal view of CD31-positive endothelial cell plexus extended toward the apical region in wild-type heart, while it was thinner and less extended in *Nkx2-5* mutant heart (Fig. 3A,B, wild-normoxia vs. mutant-normoxia, black arrowheads). Quantitative measurement of the CD31-positive area relative to the total ventricular area showed that it was lower in *Nkx2-5* mutant hearts compared to the control wild-type hearts at normoxic conditions with a *P* value of 0.068 (Fig. 3B, wild-normoxia vs. mutant-normoxia). Of note, we eliminated the peripheral ventricular area for measurement due to high background on the images generated during photo capturing.

Intermittent hyperoxia made CD31-positive coronary vessel organization significantly thicker and more extended relative to normoxic conditions in *Nkx2-5* mutant hearts (Fig. 3A,B, mutant-normoxia vs. mutant-hyperoxia, black arrowheads). In addition, the compact layer was thicker in E12.5 mutant-hyperoxia hearts relative to the mutant-normoxia hearts with *P* value below 0.05 in the right ventricle and with *P* value of 0.054 in the left ventricle (Fig. 3C,D). Overall, there was a correlation between coronary vessel organization and compact layer thickness in E12.5 *Nkx2-5* mutant hearts.

### Increase in Nkx2-5 protein expression by hyperoxia

**Figure 4:**
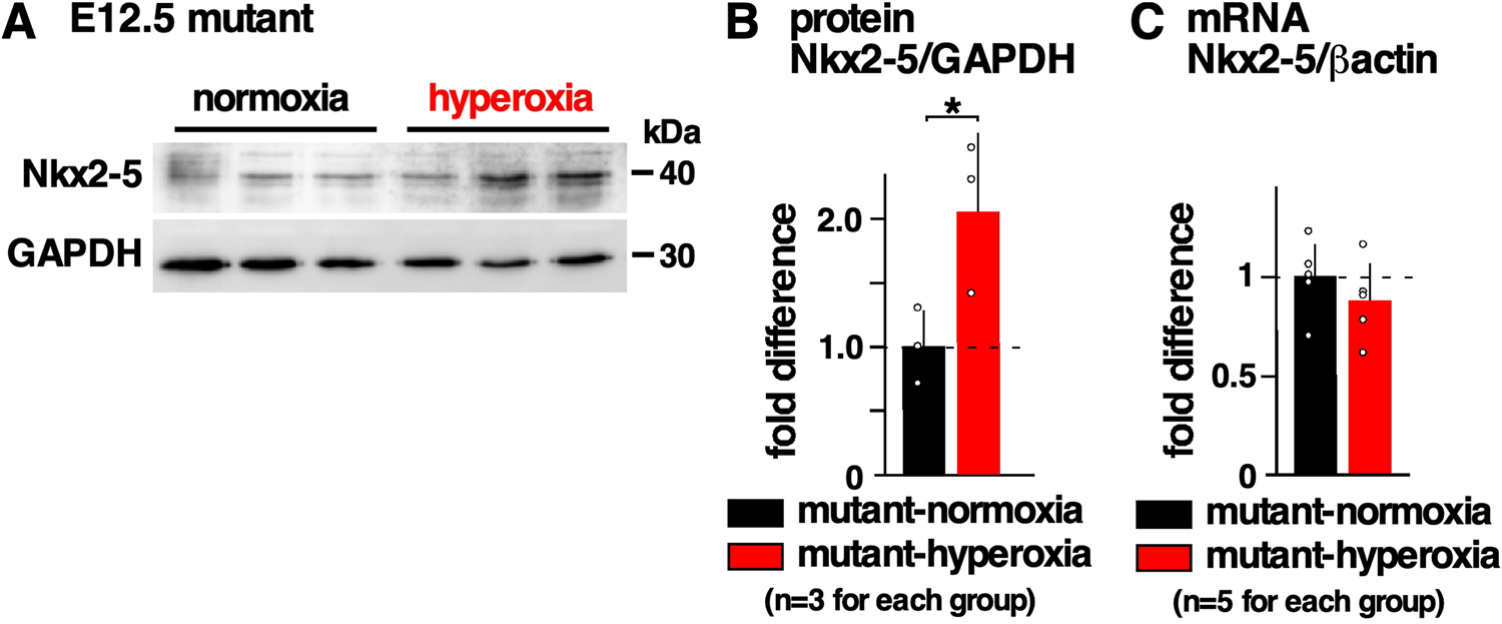
Expression of Nkx2-5 protein in E12.5 *Nkx2-5*^+*/R52G*^ embryonic hearts with or without hyperoxia. (**A**) Representative Western blotting demonstrating Nkx2-5 and GAPDH proteins (see Supplemental Fig. S1). (**B**) Quantitative data for Nkx2-5 protein expression relative to GAPDH. (**C**) Real-time reverse transcriptase PCR of Nkx2-5 mRNA relative to ß-actin. The value in the *Nkx2-5* mutant-normoxia was defined as 1. The Mann–Whitney U-test was used for analysis. Data are expressed as mean ± S.D.; individual data are represented as circles. *P ≤ 0.05. ImageJ 1.52d (http://imagej.nih.gov/ij) was used for analysis.

In our previous studies, we reported that an expression of total Nkx2-5 proteins is the same between *Nkx2*-5 mutant and wild-type embryos^11^, and Nkx2-5 proteins were reduced by approximately one-half under gestational hypoxia^18^. Mild hyperoxia, in contrast, increased the expression of Nkx2-5 in E12.5 mutant hearts by approximately 2.08 ± 0.34-fold relative to normoxic conditions (Fig. 4A,B). However, there was no change in Nkx2-5 mRNA between the two groups similar to the hypoxic conditions (Fig. 4C), suggesting that Nkx2-5 protein expression is regulated post-transcriptionally, consistent to the previous studies^18,36^.

## Discussion

Hyperoxygenation treatment for pregnant women with a fetal diagnosis of congenital heart disease has been studied and there has been some success in improving cardiac growth (reviewed in Co-VU et al.^20^). We applied intermittent mild hyperoxia (40% O_2_, 10 h per day) to wild-type pregnant mice with the expectation of having fetuses with genetic congenital heart disease induced by an *Nkx2-5* mutation from gestational day 8.5 to birth. The frequency of muscular VSD found in the heart and excessive trabeculation were significantly reduced by gestational hyperoxia compared to normoxic conditions. In addition, defective coronary vessel organization in *Nkx2-5* mutant embryonic hearts was improved by intermittent mild hyperoxia. These data suggest that gestational hyperoxia rescues genetic cardiac anomalies to some degree with statistical significance, but not completely. Non-genetic factors that aggravate or induce cardiac anomalies were relatively well documented^9,12^, but factors that reduce or cure such anomalies are not well studied.

According to a recent review study by Co-Vu et al., maternal hyperoxygenation therapy is practical, safe and effective in fetuses with congenital heart disease, especially in the growth of hypoplastic left heart structures^20^. In these studies, therapies were applied during the second and third trimester, in contrast to the current hyperoxia therapy, which was applied beginning from gestational day 8.5, which translates to human gestation at 2.5–3 weeks. Among nine studies reviewed, only one used a chronic hyperoxygenation therapy protocol with 8–9 L/min 100% oxygen for over 8 h per day (excluding time for bathing, meals, and work) by wearing a non-rebreather mask beginning from an average 29.6 weeks to delivery^37^. The treatment appeared feasible and improved aortic and mitral annular growth relative to the control group without statistical significance due to the low number of participants (n = 9 each). Regarding rescue of left ventricular hypoplasia by maternal hyperoxygenation, the timing of hyperoxia therapy is important; it is effective between 31 and 36 weeks of gestation, but not between 20 and 26 weeks^38^. Clinical trials with more participants will be necessary to demonstrate statistical significance for the hyperoxygenation treatment.

Human conception occurs 2 weeks before an expected period, thus appropriate pregnancy tests should be positive on the first day of a missed period^39,40^. In the clinical setting, it is possible for parents to know their genetic abnormalities in the *Nkx2-5* gene through genetic counseling. Therefore, the application of intermittent hyperoxia therapy beginning from human gestation 2.5–3 weeks might be feasible in the case that a woman actively trying to conceive starts the therapy when she misses an expected period and confirms her human chorionic gonadotropin-positive pregnancy test.

After testing feasibility and resolution of micro-CT imaging that shows comparability to traditional serial paraffin-tissue sectioning, we used micro-CT scanning with 4-µm resolution to assess cardiac anomalies in P1 mice. Scanning time for 4-µm resolution was around 30 min for one P1 heart, and it could be shorter to achieve lower resolutions, such as at 25–50 µm^41^. An additional soft-tissue contrast agents (Lugol’s solution) was used in this study to enhance contrast and resolution^41^. We not only reduced the time and effort to examine cardiac anomalies, but analyzed the images interpolated into a 3D reconstruction from multiple directions as shown in the Supplemental Video S1. 3D reconstructed imaging from serial paraffin-embedded tissue sectioning is achievable; however it requires extensive time and technical skills^11,42^. The inclusion of quantitative image analyses, such as that using Osirix software^43^, will be considered in the future.

In *Nkx2-5* mutant embryonic hearts, we found defective coronary vessel organization compared to the wild-type, for the first time to our knowledge, which was improved by gestational hyperoxia at E12.5. Although angiogenesis is generally thought to be induced by hypoxia^44–46^, coronary vessel organization in embryonic hearts was opposingly made defective by gestational hypoxia^47^. In addition, gestational hyperoxia improved coronary vessel organization in *Nkx2-5* mutant hearts. We speculate that improved coronary vessel organization helped compact layer thickening and modified trabecular layer structure in *Nkx2-5* mutant hearts. Considering that the ventricular septum is itself formed by compaction of the trabecular layer^29^, this offers an obvious correlation between the noncompaction and the multiple muscular defects in *Nkx2-5* mutant hearts^11^, both of which were improved by hyperoxia treatment.

Gestational hyperoxia was applied at a single concentration (40% O_2_) beginning on gestational day 8.5. We could not measure blood oxygen levels in pregnant mothers or fetuses housed in the hyperoxia chamber, or test the different oxygen concentrations. The timing of hyperoxia application was tested in pilot studies beginning on E8.5 (n = 22) or E10.5 (n = 7), and E8.5 appears more effective for ventricular compaction from the micro-CT imaging without statistical analyses. Different oxygen concentrations as well as the timing of exposure need to be investigated in the future. To quantitate the level of Nkx2-5 protein, we loaded the same amount of protein and normalized it with GAPDH expression to eliminate loading errors using standard methodology. In adult mouse hearts, however, the expression of house-keeping proteins, such as GAPDH, tubulin, and actin is altered by 28-day hyperoxia (30% O_2_) exposure^48^. This must be considered when analyzing a level of protein expression relative to GAPDH.

There are several additional limitations in this study. First, the mechanism by which mild hyperoxia improves cardiac anomalies in *Nkx2-5* mutants is not clear. Second, the present study lacked information regarding the details of defective coronary vessel organization in *Nkx2-5* mutants. Those include stage-dependent changes, underlying mechanisms regarding how a mutation in the *Nkx2-5* gene affects vascular developments despite Nkx2-5 not being expressed in endothelial cells or smooth muscle cells^49^, and how it is improved by hyperoxia. Third, the direct correlation between defective coronary vessel organization and cardiac anomalies including ventricular non-compaction in *Nkx2-5* mutants is not clear.

In summary, we report a potential therapy using mild gestational hyperoxia for genetic cardiac malformation induced by an *Nkx2-5* mutation.

## Materials and methods

The study was carried out in accordance with relevant guidelines and regulations, including compliance with the ARRIVE guidelines.

### Animal models with hyperoxia

The animal models were reported in our previous studies^11,18^. In short, wild-type 129/Sv female mice were placed in a hyperoxic chamber (COY Lab Products, Grass Lake, MI) that was connected to nitrogen and oxygen gas. The oxygen content was gradually increased from 20.9 to 40% over 15 min and the carbon dioxide was absorbed by Carbolime (AliMed, Inc., Dedham, MA). The cages were kept for 10 h, and returned to the normoxic condition for 14 h daily. On P1, hearts were examined for cardiac anomalies. At gestational day 12.5, mice were sacrificed immediately after moving them from the hyperoxic chamber to maintain hyperoxic conditions. All animal experiments were performed with approval from the University of Florida Institutional Animal Care and Use Committee.

### Micro-CT imaging

To stop heartbeat at diastole, hearts were soaked in 10% KCl/PBS immediately after dissection, and then fixed with 4% paraformaldehyde overnight at 4 °C, washed with PBS and kept in 70% ethanol. Two days before imaging, hearts were rehydrated with PBS, stained with iodine solution (2.5% KI and 1.3% I^2^ in ddH_2_O) overnight, and then washed with PBS following the protocol^41^. Images were obtained using GE Phoenix V|TOME|X M 240 Micro-CT (GE Sensing and Inspection Technologies GmbH, Wunstorf, Germany, Nanoscale Institute, University of Florida, Gainesville, FL). By stacking cross-sectionally sliced images, 3D X-ray images of neonatal mouse hearts were generated and analyzed by the Dragonfly program (Object Research Systems, Quebec, Canada) and ImageJ.

Digitalized images were used for counting the number of VSD, as well as for measuring the total area, the interventricular septum, and trabecular and compact layers. The number of VSD per heart were examined in whole hearts from multiple directions. A longitudinal section showing the largest left and right ventricular cavity was selected from the 3D images for measuring the total area, the interventricular septum, and trabecular and compact layers. The border between trabecular and compact layers was somewhat difficult to determine; we used the morphological border with the deepest endomyocardial recesses at the diastolic phase^50^, as described in our previous studies^11,18,42,51^. Just as importantly, some of our previous studies include a genetic marker for trabecular layers that we analyzed using atrial natriuretic factor-lacZ transgenic mice^51^.

### Histological analysis and Western blotting

Serial paraffin-embedded tissue sectioning of 5 µm thickness was used for histological analysis and immunostaining as described^11^. Immunostaining and Western blotting were performed with the following primary antibodies: CD31 (CM303, Biocare Medical, Pacheco, CA), Nkx2-5^49^, and troponin T (T6277, Sigma, St. Louis, MO). For whole-mount CD31 staining, after incubation with anti-CD31 antibody at 4 °C overnight, Rat-on-Mouse AP-Polymer (RT518, Biocare Medical, Pacheco, CA) was applied and visualized with Liquid Permanent Red (K0640, Agilent Dako, Santa Clara, CA). Whole-mount images were obtained with SMZ800 (Nikon, Melville, NY) attached to a CCD camera, and fluorescent microscopic images were obtained using Axiovert200M (ZEISS, Oberkochen, Germany). Digitalized images from tissue-staining were used for measurement with Image J software as described previously^42,47^.

### Real-time reverse transcriptase (RT)-PCR

RT-PCR was performed using inventoried TaqMan gene expression assays, Nkx2-5 Mm00657783_m1 (Applied Biosystems, Foster City, CA), and normalized to ß-actin expression (no. 4352933E). Duplicate experiments were averaged.

### Statistical analysis

Results were analyzed by SPSS (version 26). Data presented are expressed as mean values plus or minus the standard deviation (S.D.). The Shapiro–Wilk normality test was used to assess normal distribution. The Mann–Whitney U test was used to compare continuous variables without normal distribution or when the sample number was small. The independent T-test was used to compare continuous variables with normal distribution. The non-parametric Kruskal–Wallis ANOVA with Dunn’s test was used for comparison of three groups in Fig. 3B. The one-sided Fisher’s exact test was used for Table 1. *P* values ≤ 0.05 were considered significant.

## Supplementary Information

Supplementary Information 1.

Supplementary Video 1.
