# Supplementary material for: Gestational intermittent hyperoxia rescues murine genetic congenital heart disease in part
Source: Sci Rep. 2021 Mar 23;11:6608. doi: 10.1038/s41598-021-85569-9 (PMC7988122; doi:10.1038/s41598-021-85569-9)
Supplement: Supplementary file 1 — Supplementary Information 1. [file 41598_2021_85569_MOESM1_ESM.pdf]

Supplemental materials

Gestational intermittent hyperoxia rescues murine genetic congenital heart disease in part

Cassandra F. Doll, Natalia J. Pereira, Mustafa S. Hashimi, Tabor J. Grindrod, Fariz F. Alkassis, Lawrence X. Cai, Una Milovanovic, Adriana Sandino and Hideko Kasahara

Supplemental Figure 1. Full-length blots used for Figure 4A.

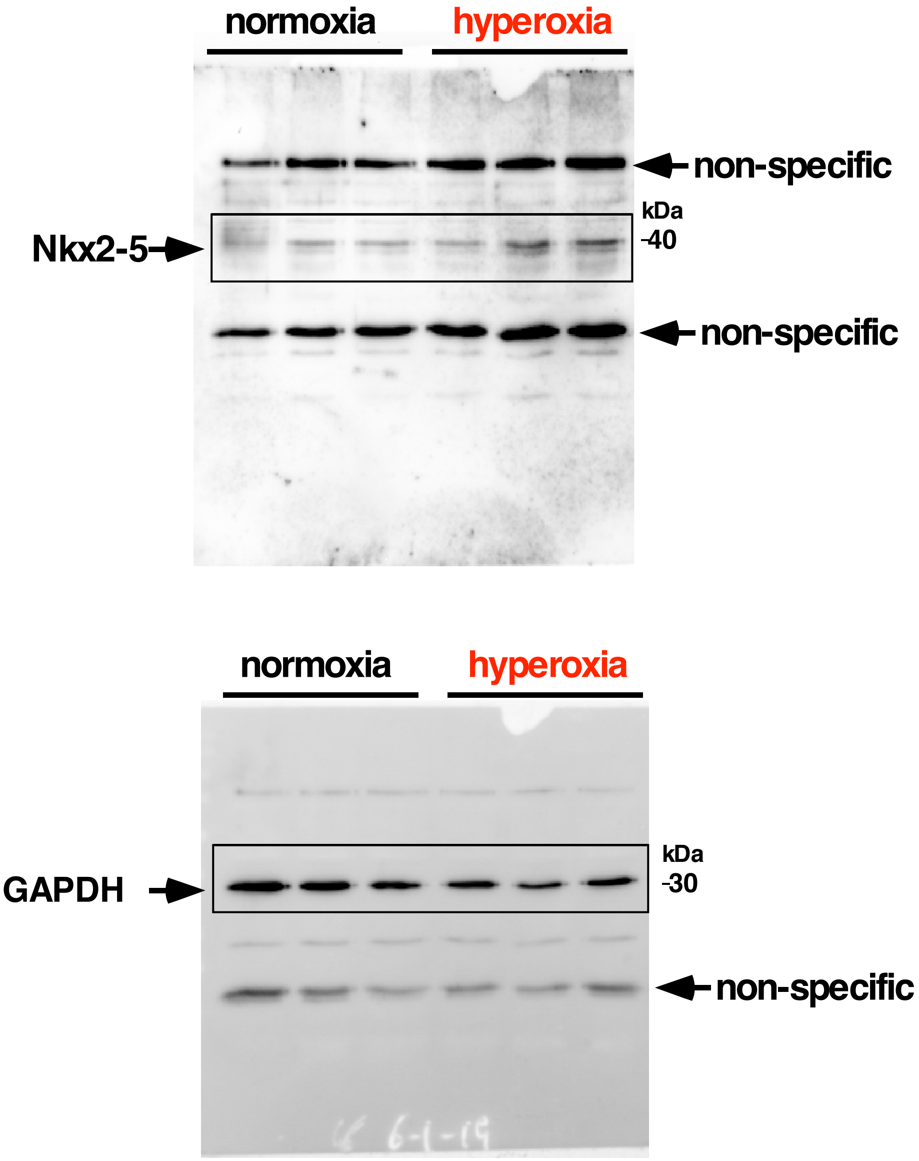

Supplemental video - analysis of microCT image
